# Theory-based physical activity and/or nutrition behavior change interventions for cancer survivors: a systematic review

**DOI:** 10.1007/s11764-023-01390-5

**Published:** 2023-05-03

**Authors:** Bruno Rodrigues, Eliana V. Carraça, Beatriz B. Francisco, Inês Nobre, Helena Cortez-Pinto, Inês Santos

**Affiliations:** 1 Centro de Investigação em Atividade Física, Saúde e Lazer (CIAFEL), Faculdade de Desporto, Universidade do Porto, Porto, Portugal; 2 Centro de Investigação em Desporto, Educação Física, Exercício e Saúde (CIDEFES), Universidade Lusófona, Lisboa, Portugal; 3 Laboratório de Nutrição, Faculdade de Medicina, Centro Académico de Medicina de Lisboa, Universidade de Lisboa, Lisboa, Portugal; 4 Centro Interdisciplinar para o Estudo da Performance Humana (CIPER), Faculdade de Motricidade Humana, Universidade de Lisboa, Lisboa, Portugal; 5 Clínica Universitária de Gastrenterologia, Faculdade de Medicina, Centro Académico de Medicina de Lisboa, Universidade de Lisboa, Lisboa, Portugal; 6 Departamento de Gastrenterologia, CHULN – Hospital de Santa Maria, Lisboa, Portugal; 7 Instituto de Saúde Ambiental (ISAMB), Faculdade de Medicina, Universidade de Lisboa, Lisboa, Portugal

**Keywords:** Systematic review, Cancer, Behavior change interventions, Physical activity, Diet

## Abstract

**Purpose:**

Theory-based interventions aimed at promoting health behavior change in cancer survivors seem to be effective but remain scarce. More information on intervention features is also needed. This review aimed to synthesize the evidence from randomized controlled trials evaluating the efficacy of theory-based interventions (and its features) on physical activity (PA) and/or diet behaviors in cancer survivors.

**Methods:**

A systematic search in three databases (PubMed, PsycInfo, and Web of Science) identified studies that (i) targeted adult cancer survivors and (ii) included theory-based randomized controlled trials designed to influence PA, diet, or weight management. A qualitative synthesis of interventions’ effectiveness, extensiveness of theory use, and applied intervention techniques was conducted.

**Results:**

Twenty-six studies were included. Socio-Cognitive Theory was the most used theory, showing promising results in PA-only trials and mixed findings in multiple-behavior interventions. Mixed findings were observed for interventions based on the Theory of Planned Behavior and Transtheoretical Model. Limited findings were found in diet-only interventions. A large variability in the extensiveness of theory use, and in intervention techniques was found. Further research is required to understand how and why these interventions offer promise for improving behavior.

**Conclusions:**

Theory-based interventions seem to improve PA and diet behaviors in cancer survivors. Further studies, including thorough intervention descriptions, are needed to confirm these findings and identify the optimal features and content of lifestyle theory-based interventions for cancer survivors.

**Implications for Cancer Survivors:**

This systematic review can contribute to the development of more effective interventions to promote long-term adherence to healthy lifestyle behaviors.

**Supplementary Information:**

The online version contains supplementary material available at 10.1007/s11764-023-01390-5.

Bruno Rodrigues and Eliana V. Carraça contributed equally to this work and share first authorship.

## Background

Improvements in early detection/diagnostics and advances in treatment are leading to an increase in the number of cancer survivors, which brings new challenges to cancer care [1]. Cancer survivors suffer from several treatment side-effects, increased risk of recurrence, and higher vulnerability to other chronic diseases [2] that may increase survivors’ risk of poor mental and physical health–related quality of life, which can be improved through modifying behavioral and psychosocial risk factors.

Lifestyle behaviors, including physical activity (PA) and a healthy diet, are key to survivorship management [3]. PA has been consistently identified as an important adjunct therapy to be incorporated in cancer care [4], given that it optimizes health outcomes [5–7], and reduces the risk of recurrence [4], mortality from cancer and any cause [5], and improves treatment’s effectiveness and tolerance [8]. Diet also plays a major role in improving health. Cancer survivors with healthier diets and adequate nutritional status have an improved treatment response/tolerance, recovery, side-effect management, and disease outcomes [9–13]. Nonetheless, and despite the beneficial effects, only a minority of cancer survivors meet PA and healthy eating recommendations [14, 15]. Moreover, even when there is good compliance at the beginning of a lifestyle behavior change program, relapse is not uncommon [16–18].

There has been a growing body of evidence trying to understand which factors or interventions facilitate adherence to lifestyle recommendations [19, 20]. However, knowledge about what works best remains scarce. Implementing theory-based interventions has been recommended to improve behavior change effectiveness [16, 21, 22]. Theory-based interventions provide a better understanding of key mediators of change, explaining why interventions might succeed or fail [16, 23], and by connecting relevant theoretical determinants of the behavior to appropriate behavior change techniques [24]. Prior research has suggested that theory-based interventions seem more effective than atheoretical approaches, and interventions combining multiple theories and targeting several constructs appear to have larger effects on improving health behaviors [16, 25]. Although appearing effective [26, 27], these interventions remain scarce [28], and target mostly short-term adherence and outcomes [29]. Furthermore, there is little consensus on the most effective theories and on which behavior change techniques are (or should be) selected to best operationalize theoretical constructs and effectively change the behavior [30, 31]. Also, in practice, the extensiveness of theory use varies substantially, precluding the assessment of its impact on behavior change [32]. In other words, little is known about how theory is applied and contributes to behavior change effectiveness.

To our knowledge, there are no systematic reviews synthesizing the effects of theory-based behavior change interventions on both PA and diet in cancer survivors with multiple types of cancer, besides one addressing only social-cognitive theory-based interventions [27]. Although there is significant evidence supporting the benefits of diet and PA on health-related outcomes, there is still insufficient information on which interventions favor sustained behavior changes in cancer survivors. Also, it has been previously noted that further investigation into the active ingredients within an intervention and the type of behavioral theory used would be useful. Therefore, this systematic review aimed to synthesize the evidence on randomized controlled trials (RCTs) evaluating the efficacy of theory-based behavior change interventions on PA and/or diet behaviors in cancer survivors. Specifically, this study seeks to (1) evaluate which theories are more effective to change PA and/or diet in cancer survivors, (2) assess the intensity of theory application in behavior change interventions, (3) investigate the relation between the extensiveness of theory use and intervention effectiveness, and (4) identify which behavior change techniques have been more often used within the interventions per theoretical framework, and if possible, which were more effective.

## Methods

This systematic review is reported in accordance with the PRISMA statement for reporting systematic reviews [33]. This review was registered in PROSPERO (registration number: CRD42021283338).

### Eligibility criteria

To be included, studies had to (i) include adults aged ≥18 years, diagnosed with any type of cancer (at any point from diagnosis and stage of disease/treatment) and (ii) report on any theory-based RCT designed to influence PA and/or diet quality, including behavioral weight management interventions, typically targeting both lifestyle behaviors. The intervention group could be compared with any parallel control group with no intervention/waiting list, usual care, or other interventions. The outcomes could be PA levels/volumes and/or diet quality and adherence.

Observational and non-intervention studies, studies with no original data, dissertations/thesis, protocols, qualitative and pilot studies and studies not published in peer-reviewed journals were excluded. Studies with children, adolescents, pharmacological or surgical interventions targeting diet and PA were also excluded.

### Search strategy

A comprehensive search of peer-reviewed articles published from inception until December 2022 (including online ahead of print publication) was conducted in three electronic databases — PubMed, PsychInfo, and Web of Science — using the following search strings: terms concerning the health condition or population of interest (e.g., Cancer, cancer survivor, cancer patient); terms concerning the intervention (e.g., Lifestyle/behavioral interventions); terms concerning the outcomes of interest (e.g., Diet, PA, weight loss/maintenance/change); and terms concerning the types of study (i.e., RCT).

A sample of the full search strategy is provided in Appendix 1. Searches were limited to English language and humans. Other searches included manual cross-referencing of literature cited in prior reviews, and hand-searches of the content of key scientific journals.

### Study selection

Titles and abstracts were screened for potential eligibility by three researchers (BBF, BR, and IN). These authors retrieved and screened the full text of potentially relevant articles. Decisions to include/exclude studies were made by consensus. When consensus was not achieved, disagreements were solved by a discussion with a fourth author (IS or EVC). The study selection procedure was conducted using the CADIMA software [34].

### Data extraction and coding

A data extraction form was developed, informed by the PRISMA statement for reporting systematic reviews [33]. Data extraction was conducted by three authors (BBF, BR, and IN) and comprised information about the article, participants, brief intervention description, used theoretical frameworks, outcomes of interest, and main findings.

The extensiveness of theory application was assessed using a modified version of Michie and Prestwich’s behavior theory coding framework [35], as done in previous studies [36]. Eight items were selected across the six categories from the original coding framework to assess the intensity of theory application, from theory and construct identification to behavior change techniques used to operationalize theoretical constructs, or measurement of these constructs. Each item was classified as present or absent based on intervention descriptions provided in the included papers or others describing the same intervention (e.g., protocols). The eight items were (1) theory was mentioned, (2) relevant constructs were targeted, (3) each intervention technique was explicitly linked to at least one theoretical construct, (4) participants were selected/screened based on prespecified criteria (e.g., a construct or predictor), (5) interventions were tailored for different subgroups that vary on a psychological construct (e.g., readiness level), (6) at least one construct or theory mentioned in relation to the intervention was measured postintervention, (7) all measures of the theory were presented with some evidence of their reliability, and (8) results were discussed in relation to the theory. One author (BR) coded this information and a second author independently checked it (EVC). Disagreements were solved by consensus. A theory extensiveness score (n/8) was then calculated [36]. A “Sparse Use of Theory” (Level 1) was considered when studies fulfilled less than 4 items. A “Moderate Use of Theory” (Level 2) was considered when studies satisfied 4 or 5 items. An “Extensive Use of Theory” (Level 3) was considered when studies fulfilled 6 or more items.

Intervention techniques were coded as present or absent using the Behavior Change Technique Taxonomy v1 [37]. An intervention technique was only coded when there was clear evidence of its direct application to PA or diet. The total number of intervention techniques used and the congruence between those and the theoretical framework underpinning each intervention was assessed. Intervention protocols and related papers were consulted when available or felt necessary. One author (BR) coded this information and a second author independently checked it (EVC). Disagreements were solved by consensus.

### Outcome measures

Total PA levels and/or PA discriminated by intensity or domains and dietary intake and/or diet quality constituted the primary outcomes of this review. Regarding PA, exercise energy expenditure (Kcal per day or week), volume (minutes per week or day), activity counts, step counts, or other measures of PA levels were considered. Concerning dietary intake, we considered caloric intake (Kcal per day or week), overall diet quality, and consumption (cup/ounces/grams/times/servings per day or week) of whole or refined grains, whole grain bread, fish, red and/or processed meat, fiber, alcohol, cruciferous, fruit, vegetables or fruit and vegetables.

### Data synthesis

Characteristics of the included studies were qualitatively synthesized and presented in tabular form, organized by outcome, type and number of theories used in each intervention. The extensiveness of theory use was also reported by outcome. A matrix crossing intervention techniques with theoretical frameworks was built and organized by outcome and global use scores.

### Study quality assessment

Study quality was assessed with an adapted version of the Quality Assessment Tool for Quantitative Studies, developed by the Effective Public Health Practice Project [38]. The current adaptation was based on recommendations from several authors [39–41] and has been previously used [39, 42]. This tool includes 19 items, organized into eight key methodological domains: study design, blinding, selection bias, withdrawals/dropouts, confounders, data collection, data analysis, and reporting. Each domain is classified as Strong, Moderate, or Weak based on specific criteria. A global rating is determined based on the scores of each component. Two researchers independently performed the quality assessment (BBF, IN). Discrepancies were resolved by consensus. When consensus was not achieved, disagreements were solved by a discussion with a third author (BR or IS or EVC). Inter-rater agreement across categories varied from moderate (Cohen’s k=0.649) to strong (Cohen’s k=1.000).

### Certainty assessment

Following the most recent PRISMA recommendations [33], the certainty of the evidence gathered in this review was assessed with the SURE checklist [43] by two researchers (BBF, IN). When consensus was not achieved, disagreements were solved by a discussion with a third author (BR or IS or EVC). This checklist includes 5 criteria to assess the identification, selection, and appraisal of studies; 5 criteria to evaluate how findings were analyzed in the review; and one criterion for other considerations. Based on the number and type of limitations identified, a conclusion regarding the degree of confidence in the evidence of a systematic review is obtained.

## Results

### Search results

**Fig. 1:**
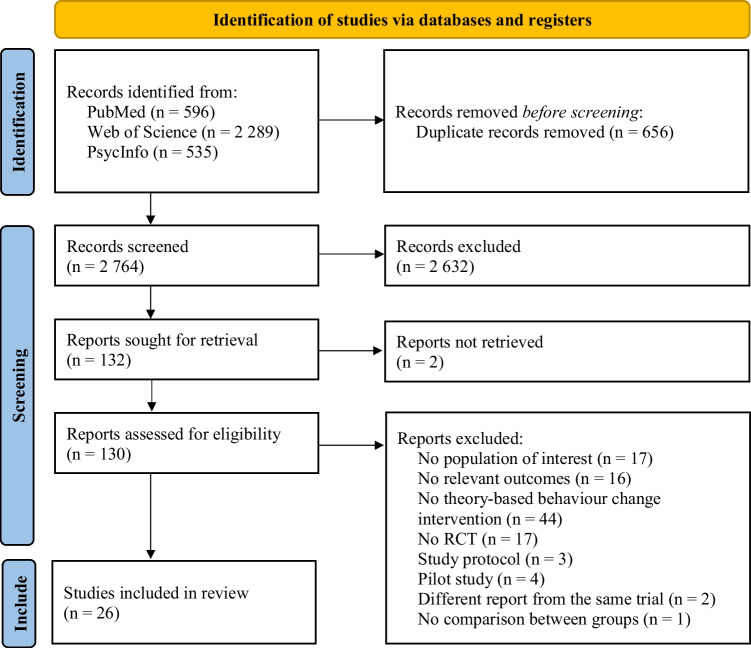
PRISMA flow diagram

Database searches resulted in 2764 potentially relevant articles after duplicates removal. Of these, 2632 were excluded based on title/abstract, leaving 132 articles for full-text screening. Twenty-six articles met the eligibility criteria and were included. Figure 1 shows the studies flow diagram.

### Studies’ characteristics

Table 1 summarizes (and Appendixes 2-4 detail) the characteristics of all included studies (*N*=26), synthesized by intervention topic: PA-only (*N*=15), diet-only (*N*=2), or multiple-behavior (PA and diet) (*N*=9).

**Table 1:** Studies’ characteristics

Characteristics	Number of studies
Sample size	
<100	11 ^45-49,51,53,55,63,65,67^
100–199	3 ^52,56,66^
200–299	6 ^50,58,59,60,68,69^
300–399	1 ^54^
≥400	5 ^44,57,61,62,64^
Participants	
Gender	
Both genders	13 ^57-69^
Men only	1 ^44^
Women only	12 ^45-56^
Mean age, years	
≥ 18*	2 ^68,69^
45–54.9	8 ^45,46,48 50-52,66,67^
55–64	12 ^44,47,49,53-56,60,61,63-65^
≥65	4 ^57-59,62^
Types of cancers	
Breast	10 ^45,48-56^
Colorectal	3 ^57–60^
Endometrial	2 ^46,47^
Prostate	1 ^44^
2 types of cancer	2 ^61,62^
Multiple cancers	7 ^63–69^
Theories used	
1 theory	15 ^44-47,49-52,60,61,65-68^
2 theories	10 ^48,53-56,58,59,63,64,69^
≥ 3 theories	1 ^62^
Outcome assessment	
< 6 months	9 ^49,51,53,54,63,65-68^
≥ 6 months	8 ^44,45,57-61,64^
Both	9 ^46-48.50,52,55,56,62,69^
Quality assessment score	
Weak	18 ^44-49,51-53,55,60-65,67,68^
Moderate	8 ^50,54,56-59,66,69^

*Participants’ age was reported in ranges (no mean age available)

Half of the studies (*N*=13) included both genders, one focused on men only [44], and twelve included women only [45–56]. The mean age ranged from 46.1 to 66.5 years. Seventeen studies focused on one type of cancer, and in this subgroup, breast cancer (*N*=10) was the most studied cancer [45, 48–56], followed by colorectal (*N*=4) [57–60], endometrial (*N*=2) [46, 47], and prostate cancer (*N*=1) [44]. Two studies included two types of cancer (breast + prostate) [61], (colorectal + prostate) [62], and seven included ≥3 cancers [63–69]. Nine studies reported short-term changes (< 6 months), eight reported changes over 6 months, and the other nine reported both short- and long-term changes (maximum 36 months length).

Most studies (*N*=15) were based on a unique theory, ten were based on two, and one on more than three theories. The most used theory was Social Cognitive Theory (SCT) (*N*=17) [44–48, 50, 53–56, 59, 61–63, 67, 69], which includes Self-Efficacy Theory (a subset of Bandura’s SCT) mentioned in one trial [49], followed by the Transtheoretical Model (TTM; *N*=7) [48, 52, 55, 56, 59, 62, 63] and the Theory of Planned Behavior (TPB; *N*=6) [51, 54, 58, 60, 68, 69]. Other theories mentioned were as follows: Health Action Process Approach (HAPA; *N*=3) [58, 62, 65], Integrated Model for Change (I-Change Model; *N*=2) [62, 64], Self-Regulation Theory (*N*=2) [62, 64], Self-Management Theory (*N*=1), Control Theory (*N*=1), Goal setting Theory (*N*=1), Acceptance and Commitment Therapy (*N*=1) [66], Health Belief Model (*N*=1) [62], and Precaution Adoption Process Model (*N*=1) [62].

Diet-only trials evaluated dietary intake/nutritional composition with interviews using a nutrition data system and a nutrient database [44] or the Dietary Screener Questionnaire (DSQ) [63].

Regarding PA-only trials, one trial used an objective measure (accelerometer) to assess PA behavior change [53], while eight relied on self-reported measures including original or adapted versions of the Seven-Day Physical Activity Recall (7-PAR), the Global Physical Activity Questionnaire (GPAQ), the Godin Leisure-Time Exercise Questionnaire (GLTEQ), the Leisure Score Index from Godin Leisure-Time Exercise Questionnaire (LSI), the Physical Activity Scale for the Elderly (PASE), the Self-reported Short Questionnaire to Assess Health-enhancing PA (SQUASH), and the Total Physical Activity Questionnaire (TPAQ) [52, 56, 60, 65–69]. All others (*N*=6) assessed PA with both subjective and objective measures (accelerometer, pedometer, or a Fitbit®).

In the nine multiple-behavior trials, two types of dietary outcomes were evaluated, using self-reported measures and questionnaires: caloric intake [45–48, 61] and dietary intake [47, 57–59, 61, 64], with one study assessing overall diet quality using the 100-point Diet Quality Index-Revised score [61]. Regarding PA, two trials used an objective measure (accelerometer) to assess PA [48, 58], while six relied on self-reported measures, original or adapted (7-PAR, LSI, SQUASH), and an interviewer administered Modifiable Activity Questionnaire [45, 46, 57, 59, 61, 64]. One RCT assessed PA with both objective (pedometer) and subjective measures [47].

### Synthesis of intervention effectiveness results

Results per outcome are detailed in Appendixes 2-4 and summarized in Table 2.

**Table 2:** Results by outcome

Outcome	Number of reports	Positive effect	Theories used	No effect	Theories used
Diet-only trials					
Dietary intake	2	2	1 SCT ^44^ 1 SCT + TTM ^63^	-	-
PA-only trials					
Objective PA	8	5	1 SCT ^50^ 1 SCT + TTM ^55^ 1 SCT + Control Theory ^53^ 1 SCT + TTM + HAPA + I-Change + … ^62^ 1 Self-Efficacy ^49^	3	1 SCT + Control Theory ^53^ 1 SCT + TPB ^54^ 1 TPB ^51^
Subjective PA	14	9	1 SCT ^50^ 2 SCT + TTM ^55,56^ 2 SCT + TPB ^54,69^ 2 TPB ^60^ 1HAPA ^65^ 1 SCT + TTM + HAPA + I-Change + … ^62^	5	1 SCT* ^67^ 1 TTM ^52^ 1 TPB ^68^ 1 Self-Efficacy ^49^ 1 Self-Management ^66^
Diet and PA trials					
Caloric intake	5	2	2 SCT ^47,61^	3	2 SCT ^45,46^ 1 SCT + TTM ^48^
Diet quality	1	1	1 SCT ^61^	-	-
Dietary intake	8	3	1 SCT ^47^ 1 SCT + TTM ^59^ 1 TPB + HAPA ^58^	5	1 SCT ^61^ 2 SCT + TTM ^48,59^ 1 ACT ^57^ 1 I-Change + SRT ^64^
Objective PA	3	3	1 SCT ^47^ 1 SCT + TTM ^48^ 1 TPB + HAPA ^58^	-	-
Subjective PA	7	4	3 SCT ^45-47^ 1 ACT ^57^	3	1 SCT + TTM ^59^ 1 I-Change + SRT ^64^ 1 SCT ^61^

*ACT*, Acceptance and Commitment Therapy; *HAPA*, Health Action Process Approach; *I-Change*, Integrated Model for Change; *PA*, Physical Activity; *SCT*, Social Cognitive Theory; *SRT*, Self-regulation Theory; *TPB*, Theory of Planned Behavior; *TTM*, Transtheoretical Model; *p-value NA

### Diet-only trials

One trial was based on SCT [44], reporting significant improvements in every aspect of dietary intake. The other trial was based on SCT plus TTM, reporting significantly lower daily servings of processed meat at 9 and 15 weeks in the intervention group (IG), but non-significant differences in fruits and vegetables and whole grain consumption [63].

### PA-only trials

Three trials used the TPB: two reported significant differences in PA from baseline to 1 year [60] and in total 3-month PA in the intervention subgroup of participants who initially reported ≤300 min/week of PA compared to the CG [68], while the other did not report significant results [51].

Of the two studies based on SCT, one reported a greater increase in minutes per week of moderate-vigorous PA (MVPA) in the intervention group (vs. controls), although not statistically tested [67], and the other reported significant improvements in objective PA at 3 months and in self-reported PA at 3 and 6 months, compared with the control group [50]. One trial was based on Self-Efficacy Theory (a subset of Bandura’s SCT), reporting significant differences in steps but not in self-reported PA between groups [49].

One trial was based on HAPA [65] and reported significant changes on self-reported PA at 4 weeks in the intervention group, but none at 14 weeks, compared to the control group. Interventions based on the Self-Management Theory [66], or the TTM [52], reported no between-group differences in subjective PA.

Six trials used a combination of different theories. Of these, five used a combination of SCT and another theory. The two interventions using SCT plus TPB showed an improvement in self-reported PA at 3 months [69] compared to the control arm. At 4 months, the tailored intervention group significantly reduced the odds of not doing any resistance-based PA, while increasing the odds of meeting resistance training guidelines [54]; no change was observed in the odds of meeting aerobic guidelines or mean daily steps. Other two trials used a combination of SCT plus TTM, showing increases in the intervention group’s (vs. controls) self-reported moderate PA [56] and in subjective and objective MVPA [55] at both 3 and 6 months, although this effect dissipated at 12 months [56]. One intervention used SCT plus Control Theory reporting significant differences in MVPA [53]. Finally, one trial [62] used a combination of multiple theories (SCT, TTM, HAPA, the I-Change Model, Health Belief model, goal setting theories, self-regulation theories and the Precaution Adoption Process Model), resulting in significant improvements in the intervention group’s self-reported MVPA and days with at least 30 min of PA at 3 months, and self-reported PA at 6 months. Objective MVPA also increased significantly in the intervention group, but objectively assessed days ≥30 min of PA was borderline significant.

### Multiple-behavior trials

Four RCTs used SCT [45–47, 61]. Of these, one reported significant improvement in every aspect of diet intake (including caloric intake) in the intervention group (vs. controls) [47], and another one showed significant differences in the total percentage of calories from fat and in diet quality, although fruit and vegetable intake did not significantly differ between groups at the 2-year follow-up [61]. The remaining two studies reported non-significant differences in caloric intake [45, 46].

Regarding PA, one study reported significant improvements in self-reported PA at 3 months, and significant differences in steps/day, LSI and PA minutes at 6 months; at 12 months there were still significant differences in LSI and PA minutes [47]. The remaining three studies reported significant differences in PA measured with the LSI at 3, 6, and 12 months [46], and for daily caloric expenditure by the end of the 12-month intervention [45]. One study reported non-significant differences between groups [61].

One trial was based on the Acceptance and Commitment Therapy [57] and did not show significant group differences in fruit, fiber, or alcohol intake. Nevertheless, the intervention group was more likely to meet Australian PA recommendations than the controls.

Several trials used combinations of theories. Two trials used SCT plus TTM. One found that daily caloric intake improved within the intervention group, but not between groups [48]. The other found inconsistent improvements in fruit and vegetable consumption were reported using a two-item screening question, but not with the Food Frequency Questionnaire [59]. One study reported no significant changes in subjective PA [59], but showed increases in objectively measured MVPA [48] at 3 and 6 months.

TPB plus HAPA were used in one trial [58], showing significant increases in the odds of consuming less processed meat at all time-points and refined grain at months 6 and 24. In the subgroup of patients who had <300 min of MVPA per week at baseline, there was not a significant improvement in the two PA outcomes. However, patients who received PA interventions had larger increases in PA at months 6 and 18 than those who did not. One study was based on Self-Regulation Theory plus I-Change Model [64], with non-significant intervention effects in either diet or PA variables.

### Extensiveness of theory use

Table 3 depicts the presence or absence of each indicator considered to calculate the extensiveness of theory use score by study and intervention target (diet, physical activity, or both). There was a vast heterogeneity across studies, but in general, the theoretical framework underpinning interventions was mentioned in all studies, and relevant constructs were targeted (except for two studies [47, 54]). On the other hand, only three studies [49, 50, 62] linked intervention techniques to at least one theoretical construct and no study selected/screened participants based on scores from a theory-related construct. Interventions were tailored to different subgroups that varied on a psychological construct in nine studies [52, 54, 56, 58, 59, 61, 62, 64, 69]. Twelve studies [44, 48, 49, 51, 55, 56, 59, 61, 63, 65, 67, 69] measured at least one construct or theory post-intervention, and 10 studies [49–51, 55, 56, 59, 63, 65, 67, 69] reported using reliable theory measures. Results were discussed in relation to theory in nine studies [49, 55, 56, 61, 63, 65–67, 69]. Overall, 15 studies [44–48, 50, 51, 53, 54, 57, 58, 60, 64, 66, 68] were classified with sparse use of theory (Level 1), 6 studies [59, 61–63, 65, 67] were classified with a moderate use of theory (Level 2), and 5 studies [49, 52, 53, 55, 56] were classified with an extensive use of theory (Level 3).

**Table 3:** Summary of the extensiveness of theory use per study

	Theoretical frameworks	1. Theory mentioned	2. Relevant constructs targeted	3. Change method linked to 1+ constructs	4. Participants selected based on theoretical construct	5. Interventions tailored to different subgroups	6. 1+ construct or theory measured post-intervention	7. Theory measures with some evidence of reliability	8. Results discussed in relation to theory	Overall intensity score
Diet-only trials										
Parsons et al., 2020	SCT	X	X				X			1^**a**^
Miller et al., 2020	SCT-TTM	X	X				X	X	X	2^**a**^
PA-only trials										
Ungar et al., 2015	HAPA+RMS	X	X				X	X	X	2^**b**^
Hirschey et al., 2018	SET	X	X	X			X	X	X	3^**b**^
May et al., 2009	SM	X	X						X	1
Rogers et al., 2014	SCT	X	X	X				X		1^**b**^
McGinnis et al., 2021	SCT	X	X				X	X	X	2^**b**^
Courneya et al., 2016	TPB	X	X							1^**b**^
Bélanger et al., 2014	TPB	X	X							1^**b**^
Vallance et al., 2015	TPB	X	X							1
Kong et al., 2021	TTM	X	X			X	X	X		3
Weiner et al., 2019	SCT-CT	X	X							1^**b**^
Webb et al., 2019	SCT-TPB	X	X			X	X	X	X	3^**b**^
Short et al., 2015a^1^	SCT	X	X	X		X	X	X		3^**b**^
Short et al., 2015b^2^	TPB	X	X	X		X				1^**b**^
Pinto et al., 2013	SCT-TTM	X	X			X	X	X	X	3^**b**^
Pinto et al., 2015	SCT-TTM	X	X				X	X	X	3^**b**^
Golsteijn et al., 2018	Multiple theories^3^	X	X	X		X				2^**b**^
Multiple-behavior trials										
Hawkes et al., 2013	ACT	X	X							1^**b**^
Sturgeon et al., 2017	SCT	X	X							1^**b**^
Gruenigen et al., 2008	SCT	X	X							1^**b**^
Mosher et al., 2012	SCT	X	X			X	X		X	2^**a**^
Gruenigen et al., 2012	SCT	X								1^**c**^
Kanera et al., 2016	ICM-SRT	X	X			X				1^**b**^
Lee et al., 2018	TPB-HAPA	X	X			X				1^**b**^
Rogers et al., 2009	SCT-TTM	X	X			X				1^**c**^
Campbell et al., 2009	SCT-TTM	X	X			X	X			2^**a**^

X: yes; blank: no

Theoretical frameworks — *ACT*, Acceptance and commitment therapy; *CT*, Control Theory; *GST*, Goal setting Theories; *HAPA*, HAPA-based counseling; *HBM*, Health Belief model; *ICM*, Integrated Model for Change; *SCT*, Social Cognitive Theory; *SET*, Self-Efficacy Theory; *SM*, Self-Management; *TPB*, Theory of Planned Behavior; *TTM*, Trans- theoretical Model; *TSR*, Theories of Self-Regulation; *PAPM*, Precaution Adoption Process Model; *SRT*, Self-Regulation Theory

Overall theory extensiveness score: 1 — Level 1, sparse use of theory; 2 — Level 2, moderate use of theory; 3 — Level 3, extensive use of theory. ^1^Short et al., 2015 — tailored-print intervention; ^2^ Short et al., 2015 — targeted-print intervention; ^3^multiple theories used: SCT-TTM-HAPA-ICM-HBM-GST- TSR-PAPM. ^a^Diet outcome (*p*<0.05); ^b^physical activity outcome (*p*<0.05); ^c^both diet and physical activity outcomes (*p*<0.05)

The relation between the extensiveness of theory use and intervention’s effectiveness was also explored (check superscript letters in the last column of Table 3). We found that interventions’ effectiveness appears to be independent of the extensiveness of theory use, given that only three interventions were ineffective in producing significant changes in the target outcomes; of these, two made sparse use of theory while the other made an extensive use; and, among effective interventions, the extensiveness of theory use varied substantially.

### Intervention techniques

Table 4 shows a matrix matching the intervention techniques used by theory or theory combination, per target outcome and overall. A total of 46 different intervention techniques – BCTs were used (32 for diet; 43 for PA) in the interventions reported in the present review. In diet interventions, the most used BCTs were goal setting behavior (used 8 times), feedback on behavior and instruction on how to perform the behavior (both used 7 times), and problem solving, social support (unspecified) and review of behavior goals (all three used 6 times). In PA interventions, the most used BCTs were goal setting behavior and self-monitoring of behavior (both used 21 times), instruction on how to perform the behavior (used 18 times), and problem solving (used 16 times).

**Table 4:** Matrix of intervention techniques by theory (or combination of theories) used

	SCT	SCT-TTM	HAPA-RMS	SET	SM	TPB	TTM	SCT-CT	SCT-TPB	SCT-TTM-HAPA…^1^	ACT	ICM-SRT	TPB-HAPA	Total per BCT
Goal Setting (behavior)^44-48,50-58,60-68^	Diet 4 PA 6	Diet 1 PA 2	PA 1		PA 1	PA 3	PA 1	PA 1	PA 2	PA 1	Diet 1 PA 1	Diet 1 PA 1	Diet 1 PA 1	Diet 8 PA 21
Problem solving^48,50,51,53-68^	Diet 1 PA 2	Diet 2 PA 4	PA 1		PA 1	PA 3		PA 1		PA 1	Diet 1 PA 1	Diet 1 PA 1	Diet 1 PA 1	Diet 6 PA 16
Goal setting (outcome)^46,47^	Diet 2 PA 2													Diet 2 PA 2
Action planning^44,45,48,50,51,53-58,60,62,64-67,69^	Diet 2 PA 2	PA 3	PA 1		PA 1	PA 2		PA 1	PA 1	PA 1	Diet 1 PA 1	Diet 1 PA 1	Diet 1 PA 1	Diet 5 PA 15
Review behavior goal(s)^44,46,48,50,52,53,56,57,61-66^	Diet 3 PA 3	Diet 1 PA 2	PA 1		PA 1		PA 1	PA 1		PA 1	Diet 1 PA 1	Diet 1 PA 1		Diet 6 PA 12
Discrepancy between behavior and goal^59,62,64,65^			PA 1			PA 1				PA 1		PA 1		Diet 0 PA 4
Review outcome goal(s)^47,60^	PA 1					PA 1								Diet 0 PA 2
Behavioral contract^52,57,60,69^						PA 1	PA 1		PA 1		Diet 1 PA 1			Diet 1 PA 4
Monitoring of behavior by others without feedback44,57	Diet 1										Diet 1 PA 1			Diet 2 PA 1
Feedback on behavior^44-47,50,52,54,56,59,61,62,64,66^	Diet 5 PA 5	Diet 1 PA 2			PA 1		PA 1			PA 1		Diet 1 PA 1		Diet 7 PA 11
Self-monitoring of behavior^44-53,55-58,60,62,64-69^	Diet 2 PA 5	PA 3	PA 1	PA 1	PA 1	PA 3	PA 1	PA 1	PA 1	PA 1	PA 1	PA 1	Diet 1 PA 1	Diet 3 PA 21
Biofeedback^46,47,49,50,52,53,55,56,58,60,66^	PA 4	PA 2		PA 1	PA 1	PA 1	PA 1	PA 1					PA 1	PA 12
Feedback on outcome(s) of behavior^47,54,60,66^	Diet 1 PA 1			PA 1		PA 1								Diet 1 PA 3
Social support (unspecified)^44,45,48,50,53,54,58,59,63,64,66,69^	Diet 2 PA 2	Diet 2 PA 2			PA 1			PA 1	PA 1			Diet 1 PA 1	Diet 1 PA 1	Diet 6 PA 9
Social support (practical)^54,60,62,65^			PA 1			PA 1				PA 1				PA 3
Social support (emotional)^49,50,62,67^	PA 2			PA 1						PA 1				PA 4
Instruction on how to perform the behavior^44-51,53-58,60,62,63,65-68^	Diet 4 PA 5	Diet 1 PA 3	PA 1	PA 1	PA 1	PA 3		PA 1		PA 1	Diet 1 PA 1		Diet 1 PA 1	Diet 7 PA 18
Information about antecedents^50^	PA 1													PA 1
Behavioral experiments^63^		Diet 1												Diet 1
Information about health consequences^45,48-50,52,54-56,58,60-64,66,68,69^	Diet 2 PA 3	Diet 1 PA 3	PA 1	PA 1	PA 1	PA 2	PA 1		PA 1	PA 1			Diet 1 PA 1	Diet 4 PA 15
Information about emotional consequences^46,54,69^	Diet 1 PA 1								PA 1					Diet 1 PA 2
Demonstration of the behavior^45,48,50,58,60,62,65,66,67^	Diet 2 PA 4	Diet 1	PA 1		PA 1	PA 1				PA 1			Diet 1 PA 1	Diet 4 PA 9
Social comparison^50,54,58,64,66,69^	PA 1				PA 1				PA 1			Diet 1 PA 1	Diet 1 PA 1	Diet 2 PA 5
Information about others’ approval^54,69^									PA 1					PA 1
Prompts/cues^45,51-54,57,60,62,64,67,68^	Diet 1 PA 2					PA 3	PA 1	PA 1		PA 1	Diet 1 PA 1	Diet 1 PA 1		Diet 3 PA 10
Behavioral practice/rehearsal^45,46,48,50,52,60,63,65-67^	Diet 2 PA 4	Diet 1 PA 1	PA 1		PA 1	PA 1	PA 1							Diet 3 PA 9
Behavior substitution^46,47,58,63^	Diet 2 PA 2	Diet 1											Diet 1 PA 1	Diet 4 PA 3
Habit formation^44,69^	Diet 1								PA 1					Diet 1 PA 1
Habit reversal^69^									PA 1					PA 1
Generalization of target behavior^44^	Diet 1													Diet 1
Graded tasks^45,46-48,50,52,53,55,58,66,69^	Diet 3 PA 4	PA 2			PA 1		PA 1	PA 1	PA 1				Diet 1 PA 1	Diet 4 PA 11
Credible source^45,48,49,50,54,57,62-64,68^	Diet 1 PA 2	Diet 1 PA 1		PA 1		PA 1				PA 1	Diet 1 PA 1	Diet 1 PA 1		Diet 4 PA 8
Pros and cons^62,64^										PA 1		Diet 1 PA 1		Diet 1 PA 2
Comparative imagining of future outcomes^49^				PA 1										PA 1
Non-specific reward^60^						PA 1								PA 1
Social reward^62^										PA 1				PA 1
Self-reward^69^									PA 1					PA 1
Reduce negative emotions^44,46,50,52,54,62,66^	Diet 2 PA 2				PA 1		PA 1			PA 1				Diet 2 PA 5
Restructuring the physical environment^44,54,55,60^	Diet 1 PA 1					PA 1								Diet 1 PA 2
Adding objects to the environment^60,63^		Diet 1				PA 1								Diet 1 PA 1
Framing/reframing^67,69^	PA 1								PA 1					PA 2
Behavior cost^45^	Diet 1 PA 1													Diet 1 PA 1
Verbal persuasion about capability^50,62,64^	PA 1									PA 1		Diet 1 PA 1		Diet 1 PA 3
Focus on past success^44,63^	Diet 1	Diet 1												Diet 2
Self-talk^49,50,53,60,62^	PA 1			PA 1		PA 1		PA 1		PA 1				PA 5
Imaginary reward^46,49,58^	Diet 1 PA 1			PA 1									Diet 1 PA 1	Diet 2 PA 3
TOTAL, per Theory (Total BCTs used)	Diet 49 (25_BCT) PA 72 (28_BCT)	Diet 16 (14_BCT) PA 30 (13_BCT)	PA 11 (11_BCT)	PA 10 (10_BCT)	PA 15 (15_BCT)	PA 32 (19_BCT)	PA 11 (11_BCT)	PA 7 (7_BCT)	PA 15 (14_BCT)	PA 19 (19_BCT)	Diet 9 (9_BCT) PA 10 (10_BCT)	Diet 11 (11_BCT) PA 13 (13_BCT)	Diet 12 (12_BCT) PA 13 (13_BCT)	Diet 97 (32_BCT) PA 261 (43_BCT)

*ACT*, Acceptance and commitment therapy; *CT*, Control Theory; *SCT*, Social Cognitive Theory; *GST*, Goal setting Theories; *HAPA*, HAPA-based counseling; *HBM*, Health Belief model; *ICM*, Integrated Model for Change; *PAPM*, Precaution Adoption Process Model; *SET*, Self-Efficacy Theory; *SM*, Self-Management; *TPB*, Theory of Planned Behavior; *SRT*, Self-Regulation Theory; *TTM*, Transtheoretical Model; ^1^multiple theories used: SCT-TTM-HAPA-ICM-HBM-GST- TSR-PAPM

SCT was the most applied theoretical framework, with the number of BCTs used in these interventions being far greater than the number of BCTs used in interventions supported by other theoretical rationales (SCT: 25 BCTs for diet and 28 BCTs for PA; other theories around 12 BCTs for diet and 13 BCTs for PA, on average). Goal setting (4 for diet; 6 for PA), feedback on behavior (5 for diet; 5 for PA), instruction on how to perform the behavior (4 for diet; 5 for PA), graded tasks (3 for diet; 4 for PA), review of behavior goals (3 for diet; 3 for PA), self-monitoring of behavior (2 for diet; 5 for PA), biofeedback (4 for PA), demonstration of the behavior and behavioral practice/rehearsal (2 for diet; 4 for PA) were the most used BCTs in SCT-based interventions.

A large heterogeneity was observed, not only across interventions based on distinct theoretical frameworks, but also in interventions based on the same rationale. Hence, no single intervention used the same combination of BCTs. In addition, several interventions were based on different combinations of theories. This prevented us from assessing which BCTs were more effective in changing behavior, and understanding why or how the theories that appeared more effective work.

### Risk of bias assessment

Results are reported in Appendix 5.

Both diet-only studies were classified with weak methodological quality [44, 63]. Of the fifteen PA-only studies, five were rated moderate [50, 54, 56, 66, 69], and ten weak [49, 51–53, 55, 60, 62, 65, 67, 68]. In multiple-behavior studies, three were rated with moderate [57–59] and six with weak quality [45–48, 61, 64].

The areas with a higher risk of bias were selection bias (all studies involved samples of volunteers) and blinding (not performed in several studies as interventions were rarely concealed from participants and/or outcome assessors). Seven studies did not control for confounders or did not describe them, being attributed a weak quality in this domain.

### Assessment of evidence’s certainty

Results are reported in Appendix 6.

The SURE checklist [43] indicated that this systematic review has important limitations. Language bias was not avoided, considering that the search was restricted to papers written in English. Therefore, a more comprehensive search could have resulted in a higher number of retrieved papers. The list of excluded studies was not provided. Results could not be combined, and heterogeneity could not be explored due to methodological differences in studies and to the scarcity of studies per theory. Nevertheless, the findings of the current systematic review can be considered informative.

## Discussion

This systematic review aimed at synthesizing the literature on theory-based behavior change interventions designed to improve PA and/or diet in cancer survivors, generally supporting their efficacy. Results indicated that diet-only interventions (although scarce) had beneficial effects on at least one aspect of diet (e.g., reducing the consumption of processed meat). Dietary changes were less consistent in multiple-behavior interventions, possibly because the primary focus of most of these trials was other than changing diet (e.g., weight reduction, improve PA or quality of life). Most multiple-behavior trials reported significant improvements in PA, as did most PA-only trials.

Regarding theoretical frameworks, SCT was the most used, followed by TTM and TPB. In diet-only interventions, SCT was used in isolation or combined with TTM, and though apparently effective, too few diet trials exist to date, demanding further exploration. PA-only trials based on SCT, used in isolation or combined with other theories, generally showed beneficial effects on PA. Our results mirror those reported in a recent meta-analysis [27], showing that SCT-based interventions resulted in meaningful changes in diet and PA behaviors in cancer survivors. This is especially true for diet-only and PA-only trials, but not so much for multiple-behavior trials, which in our systematic review led to mixed findings, possibly due to the different theoretical combinations, variability in the extensiveness of theory use, and large heterogeneity in the employed intervention techniques.

In line with prior research [70–73], interventions based on TPB and TTM led to mixed findings, with some studies pointing towards positive changes in the outcomes of interest, while others could not find significant effects. Additional studies are required to validate the efficacy of both these theories, especially in the field of nutrition/diet. On the other hand, HAPA-based interventions showed promising results, consistent with those of a pilot trial testing a HAPA-based intervention, which found significant increases in the frequency of breaks from sitting in full-time university students [74]. However, HAPA’s use is still limited and rarely in isolation. More interventions are thus required to confirm these findings.

Other theories were seldom used, making it difficult to draw conclusions about their effectiveness. Interestingly, no interventions based on Self-Determination Theory were found, though prior research has shown that internal motivations play an important role in long-term, sustained, behavior adoption [75–77], supporting its use as a valid framework.

The extensiveness of theory use in the interventions included in this review might explain the observed mixed findings. We found that more than half of the interventions made a sparse use of theory, rarely linking intervention techniques to theoretical constructs, selecting participants or tailoring interventions to subgroups based on psychological constructs. Measurement and interpretation of results in relation to theory were also inconsistent across studies. These results suggest that theory use was rather insubstantial, alerting once again for the necessary distinction between theory-informed interventions and theory-based interventions [78–80]. Studies included in this review were often not explicit about how the theory was operationalized, i.e., by specifying which theory-relevant determinants of behavior were targeted and how (through which intervention techniques). Interventions truly based on theory should include these aspects in their design [81, 82].

According to our findings, interventions’ effectiveness seems to be independent of the intensity of theory use. One aspect that might explain these results is the large variation in the target outcomes. Several and distinct dietary measures were used in the studies, as happened with PA measures. Every time an intervention presented a significant change in one of these measures, it was considered effective in the present review. We did/could not match effects by identical measures, which might have had an influence in these results. Also, besides the large variability in the extensiveness of theory use, the combinations of theories used varied substantially, preventing us from making more solid conclusions. Finally, we cannot discard the hypothesis that interventions with an extensive use of theory could turn out to be more effective in the long-term, throughout life. Most of the studies included in this systematic review reported on changes in diet and/or PA in the first 12 months (or less) following the intervention. It is known that times of adversity, like a cancer diagnosis, create a unique set of circumstances for behavior change whereby patients are met with a “teachable moment” [83]. Indeed, after active treatment completion, over half of cancer survivors are willing and feel able to participate in exercise [18] and report an increase in the number of health-related goals [84]. It is thus possible that in the long run, we could observe clearer distinctions in the outcomes of these interventions, perhaps greater in those interventions using theory more extensively, or using a certain theory or combination of theories.

The most used intervention techniques in the trials included in this review, and targeting changes in PA and/or diet, were goal setting of behavior, self-monitoring of behavior, feedback on behavior, instruction on how to perform the behavior, and problem solving, in line with previous systematic reviews in chronic disease populations [85, 86]. Still, a large heterogeneity was observed, not only across interventions based on distinct theoretical frameworks, but also in interventions based on the same rationale. No consistent (theory-congruent) combinations of techniques for improving PA or diet in this population were identified. In addition, interventions were based on varied combinations of theories. This prevented us from assessing which intervention techniques were more effective in changing behavior, or how the theories appearing more effective did work. The combination of techniques used might potentiate or suppress the effect of certain BCTs used in isolation, due to putative interaction effects, also depending on contextual factors and other intervention characteristics [87]. Moreover, interventions were not always described in sufficient detail, possibly leading to the inappropriate (mis) identification of BCTs in some of the interventions included in the current review. This issue has been previously observed [87–89]. The way the planned intervention is implemented afterwards might also influence its effectiveness, depending on the style of delivery or on the appropriateness of implementation of the selected techniques [90, 91].

This is the first review summarizing the evidence of RCTs evaluating the effects of multiple behavior change theories on both PA and/or dietary patterns in cancer survivors with multiple types of cancer, providing a broad overview of existing theory-based interventions in this population.

### Limitations

The present systematic review has several limitations that should be considered when interpreting the results. A broad definition of cancer survivors was considered herein, increasing the evidence’s breadth but contributing to increased heterogeneity across studies. The limited number of trials targeting diet only did not allow us to withdraw any conclusions regarding this type of intervention. PA outcomes were predominantly based on self-reported data and most interventions were unsupervised. Only two trials focused on promoting resistance-training, despite current PA recommendations [5], which precluded the exploration of effects across different PA types. More than two thirds of the studies were rated as “weak quality,” calling for improvements in research methodologies. Results could not be combined and quantitatively summarized due to methodological differences and to the scarcity of studies per theory (or combination of theories). Most interventions used theory sparsely, suggesting they were theory-informed rather than truly theory-based. Also, no consistent (theory-congruent) combinations of techniques for improving diet and/or PA were identified in this population. These findings call for more detailed descriptions of theory operationalization (which constructs were targeted and through which specific intervention techniques), greater standardization in the identification of employed intervention techniques (by using comprehensive taxonomies), assessments of the style of delivery and measures of implementation fidelity, which are clearly required and likely to have an impact on the determination of theory-based interventions’ effectiveness. Comparing the effectiveness of interventions using different theories through meta-analysis and assessing whether single or multiple health behavior interventions have the greatest benefit to improve health behaviors would also be useful gaps to address.

### Clinical implications

Our review suggested that theory-based interventions are important to improve and maintain PA and diet behaviors in cancer survivors, but which theories or combinations of theories are more effective and why requires further investigation. Nevertheless, the available evidence seems to support the use of theory-based interventions, if carefully designed and planned. Clearly identifying which theoretical constructs will be targeted, through which intervention techniques, and how they will be measured is recommended if professionals are to truly understand how to effectively change the target behavioral outcomes, PA and diet,﻿ in cancer survivors.

## Conclusions

This systematic review suggests that theory-based interventions seem important to improve PA and diet behaviors in cancer survivors. SCT was the most used theory, showing promising results in PA-only trials. Mixed findings were found for multiple-behavior interventions based on SCT, and for interventions based on TPB or TTM. Limited but potentially favorable findings were found in diet-only interventions and in HAPA-interventions. We found a large variability in the extensiveness of theory use and in the employed intervention techniques. Therefore, further research is required to corroborate our findings and understand how and why these interventions offer promise for improving behavior. This information can contribute to the development of more effective interventions to promote adherence to healthy lifestyle behaviors. Notwithstanding the beneficial effects of the theory-based interventions included in this systematic review, more research is needed to identify the optimal features and content of lifestyle interventions for cancer survivors.

## Supplementary information

ESM 1

## Data Availability

Being a systematic review paper, all data is available on the articles included in the review. The full data extracted from the articles can be found in Appendixes 2-4.
