# Supplementary material for: Theory-based physical activity and/or nutrition behavior change interventions for cancer survivors: a systematic review
Source: J Cancer Surviv. 2023 May 3;18(5):1464–80. doi: 10.1007/s11764-023-01390-5 (PMC11424668; doi:10.1007/s11764-023-01390-5)
Supplement: Supplementary file 1 — ESM 1 [file 11764_2023_1390_MOESM1_ESM.doc]

Appendix 1 - Search Strategy

1) Diet* OR eating OR nutrition OR “nutrition therapy” OR “lifestyle intervention” OR “dietary intake" OR food

2) “Weight management” OR “Weight control” OR “Weight loss” OR “Weight maintenance”

3) training OR “Physical activity” OR exercise OR walking OR aerobic

4) ("behaviour intervention" OR "behavior intervention" OR "behavioral intervention" OR “behavioural intervention” OR “behaviour therapy” OR “behavior therapy” OR “behavioral therapy” OR “behavioural therapy” OR “behaviour program” OR “behavior program” OR “behavioral program” OR “behavioural program” OR “behaviour change” OR “behavior change” OR “behavioral change” OR “behavioural change”

5) Cancer OR “cancer survivor*” OR “cancer patient”

6) RCT OR “randomised controlled trials” OR “randomised controlled trial”)

7) 1) OR 2) OR 3)

8) 5) AND 4) AND 6) AND 7)

Appendix 2 – Diet-only Trials

| **Authors** | **Cancer Type/Phase** | **Sample** | **Intervention** | **Theory** | **Outcomes** | **Results** |
| --- | --- | --- | --- | --- | --- | --- |
| Parsons et al., 2020 | Prostate (stage < cT2) with no prior prostate cancer treatment with surgery, radiation, local ablation, or androgen deprivation therapy or metastatic disease; | Overall: 443;  IG: 226, 0% women, 63,7 ± 6,5 years;  CG: 217, 0% women, 63,5 ± 6,6 years; | IG: 4 phases telephone counselling intervention: The first phase (6 counselling telephone calls over 1month) focused on building self-efficacy; the second (4 calls over 2 months) on consolidating the new dietary pattern; the third (4 calls over 4 months) on relapse prevention; and the fourth (8 calls over 16 months) on providing positive feedback and monitoring for declining interest.  CG: Printed materials from the Prostate Cancer Foundation encouraging consumption of a vegetable-rich diet.  24 months | - Social Cognitive Theory | Diet composition (Interviews using the Nutrition Data System for Research software and nutrient database)  Baseline, 12-month follow-up, and 24-month follow-up. | At 12-month follow-up, intervention participants reported significant increases compared with controls in daily total vegetable servings (mean change 2,43 vs. 0,45; p < 0.001) and cruciferous servings (mean change, 43.10 g/d vs 6.44 g/d; p < 0.001).  At 24-month follow-up, significant between-group differences persisted for total vegetable servings (mean change, 2.01 vs 0.37; p < 0.001) and cruciferous servings (mean change, 0.50 vs 0.01; p < 0.001). |
| Miller et al., 2020 | Multiple cancers at any stage, who completed active cancer treatment (Breast 47%, Metastatic breast 11%, Blood 9%, Female reproductive 8%, Multiple cancers specified 11%, Other 13%) | Overall: 53, 92% women, 61,2 ± 10,5 years;  IG: 26, 92% women, 59,5 ± 9,7 years;  CG: 27, 93% women, 62,8 ± 11,1 years | IG: Eight, in-person, 90-min group meetings convened weekly at community-based organizational facilities with: (1) Nutrition Education (2) Structured Group Learning and Support (3) Cooking Demonstration + Sharing and Caring Potluck + recipe cards + workbook + S.M.A.R.T Goal-Setting Worksheets  CG: CCK printed educational materials (7 written summaries of weekly nutrition content and 14 recipes that emphasized the weekly nutrition themes)  8 weeks intervention + 15 weeks follow up | - Social Cognitive Theory;  - Trans theoretical Model | Dietary Intake (Dietary Screener Questionnaire (DSQ) in the NHANES 2009-10):  - Total Fruit and Vegetable, cup equivalents per day  - Whole Grains Total, ounce equivalents per day  - Processed Meat, times per day  (1/2 cup equivalent fruit and vegetable: 1 daily serving. A total of 0.56 oz equivalent whole grains: 1 daily serving.)  Baseline, post-intervention (9 week), and at follow-up (15 week) | There were no statistically significant differences in fruits and vegetables and whole grain consumption between groups.  The intervention group had significantly lower daily servings of processed meat in comparison to the control group at 9 weeks (mean 0,04±0,05 vs 0,10±0,18) and 15 weeks (mean 0,04±0,05 vs 0,11±0,17; p < 0.05). |

CG: Control Group; IG: Intervention Group;

Appendix 3 – Multiple-Behaviour Trials

| **Authors** | **Cancer Type/Phase** | **Sample** | **Intervention** | **Theory** | **Outcomes** | **Results** |
| --- | --- | --- | --- | --- | --- | --- |
| Hawkes et al., 2013 | Colorectal at any stage/phase of treatment | Overall: 410;  IG: 205, 48,3% women, 64,9 ± 10,8 years;  CG: 205, 43,9% women, 67,8 ± 9,2 years; | IG: Telephone-delivered health coaching intervention (11 sessions) + participant handbook + regular motivational postcard prompts + a pedometer + newsletter  CG: usual care - educational brochures produced by Cancer Council Australia on understanding CRC and cutting cancer risk, diet, and PA.  6 months | - Acceptance and commitment therapy | - Dietary intake (Cancer Council Victoria Food Frequency Questionnaire)  - PA (modified Leisure Score Index (LSI) from Godin Leisure-Time Exercise Questionnaire (GLTEQ))  Baseline, 6 and 12 months | - No significant group differences were found at 6 or 12 months for fruit, fibre, or alcohol intake (p>0.05).  - Compared with the control group, the intervention group was also more likely to meet Australian PA recommendations (p = 0.047). |
| Sturgeon et al., 2016 | Breast cancer survivors (BRCA1/2+) who underwent prophylactic oophorectomy and are now cancer free | Overall: 35, 100% women, 46,1 ± 4 years;  IG: 16, 100% women, 47,2 ± 3,8 years;  CG: 19, 100% women, 45,1 ± 4 years: | IG: web-based program with 3 daily activities: workout—completing relative and progressive strength-training and aerobic exercises; habit—completing a nutritional/lifestyle habit (new habits followed every 2 weeks; and lesson — reading lessons on health, nutrition, fitness, or behaviour change.  CG: Waitlist  12 months | - Social Cognitive Theory | - Caloric intake (self-reported logging on 3-day dietary records);  - Caloric expenditure (interviewer administered Modifiable Activity Questionnaire)  Baseline and after the 12-month intervention (follow-up). | - There was no significant difference for caloric intake between groups.  - There was a significant difference between group difference for daily caloric expenditure with the intervention group increasing PA more by the end of the intervention period (740,6±330,2 vs 425,2±325,6; p = 0.04). |
| Gruenigen et al., 2008 | Endometrial (stage I-II), who had a total abdominal hysterectomy, and bilateral salpingo-oophorectomy | Overall: 45;  IG: 23, 100% women, 54 ± 2,0 years;  CG: 205, 100% women, 55,5 ± 1,6 years; | IG: 6-month individual counselling + newsletter + pedometer  CG: usual care - informational brochure  6 months | - Social Cognitive Theory | - Quantitative dietary intake: three-day food records  - PA (LSI from the GLTEQ)  Baseline, 3, 6 and 12 months | - There were no significant differences for kilocalories intake (p>0.05).  - There was a significant difference in PA between the intervention group and usual care at 3 months (mean group difference = 19.6, 95% CI = 2.5 to 36.7; p=0.025), 6 months (mean difference = 13.6; 95% CI = 1.8 to 25.3; p=0.038) and 12 months (mean difference = 15.8; 95% CI = 4.5 to 27.0; p= 0.007). |
| Mosher et al., 2012 | Early stage (in situ, localized, or regional) Breast or Prostate cancer | Overall: 489, 62,58% women, 57,2 ± 10,7 years; | IG: tailored materials designed to increase fruit and vegetable (F&V) intake, decrease fat intake, and/or increase PA.  CG: mailed print materials on diet and PA available in the public domain  10 months | - Social Cognitive Theory | - Diet: Number of servings of F&V per day, percentage of kcal from fat (The Diet History Questionnaire (DHQ)), and diet quality, (100-point Diet Quality Index-Revised score)  - PA: Total minutes per week of MVPA (7-day Physical Activity Recall (7-day PAR));  Baseline, 2-year follow-up | - F&V intake and PA did not significantly differ between groups at 2-year follow-up.  - Diet Quality Index-Revised score (IG: 71,5±10,5 vs CG: 68,9± 10,6; p<0.001),  - and total percent of calories from fat (IG: 36,5±6,6 vs CG: 37,8±5,6; p=0.001) were significantly different between groups.  - PA did not significantly differ as a function of group assignment |
| Gruenigen et al., 2012 | Endometrial (stage I-II), after a total abdominal hysterectomy, bilateral salpingo-oophorectomy | IG: 41, 100% women, 57,0 ± 8,6 years; CG: 34, 100% women, 58,9 ± 10,9 years; | IG (SUCCEED intervention): Physician face-to-face counselling + feedback and support via newsletters, telephone and email + pedometer  CG: usual care - informational brochure: “Healthy Eating & Physical Activity Across Your Lifespan, Better Health and You”  6 months | - Social Cognitive Theory | - Dietary intake: fruit/vegetable servings/day, kilocalories (Nutrition Data System for Research Software (NDSR) versions 2008 and 2009)  - PA minutes (LSI from the GLTEQ); calculated as the number of moderate minutes plus two times vigorous minutes.  Diet and PA minutes: Baseline, 3, 6, 12 months  - Pedometer step count was assessed at baseline and 6 months for a one-week period and an average daily count was calculated. | - Fruit intake only had positive significant differences at 12 months (IG: 2,1 ± 2,5 vs CG: 1,8±1,8; p=0.032).  - Vegetables intake had positive significant differences at 3 (IG: 4,2±2,9 vs CG: 3,0±2,1; p<0.001), 6 (IG: 4,0±2,5 vs CG: 3,2±2,4; p=0.001) and 12 months (IG: 3,7±2,5 vs CG: 3,4±2,0; p=0.004).  - F&V had positive significant differences at 3 (IG: 6.2±3.5 vs CG: 5.0±2,5; p<0.001), 6 (IG: 5,9±3,2 vs CG: 4.9±3.0; p<0.001) and 12 months (IG: 5,6±3,6 vs CG: 5,0±2,4; p<0.001).  - Kilocalories intake had positive significant differences at 3 (IG: 1553,2±448.4 vs CG: 1723,8±533,3; p<0.001), 6 (IG: 1635,2±579,2 vs CG 1844,6±624,5; p<0.001) and 12 months (IG: 1606,6±495,5 vs CG: 1806,9±631,6; p<0.001)  - There were positive significant differences in steps per day (p=0.015) between groups at 6 months. Mean change from baseline to 6 months was 2353 in the IG group versus −9.4 steps/day in CC [difference (95% CI) of 2362 (494, 4230); p=0.015].  - No significant differences in PA minutes at 3 months, but positive significant differences between groups at 6 (IG: 249±227 vs CG: 144±180; p=0.038) and 12 months (IG: 216±190 vs CG: 142±128; p=0.020).  - Positive significant differences in leisure score index at 3 (IG: 39,5±31,5 vs CG: 28,4±20,4; p<0.001), 6 (IG: 33,0±23,5 vs CG: 20,9±18,6; p<0.001), and 12 months (IG: 30,7±21,1 vs CG: 23,4±18,9; p<0.001). |
| Rogers et al., 2009 | Breast (stage I-IIIA), receiving hormonal therapy | Overall: 41, 100% women, 53 ± 9 years;  IG: 21, 100% women, 52 ± 15 years;  CG: 20, 100% women, 54 ± 8 years; | IG: 6 discussion group sessions with a clinical psychologist + 12 individual exercise sessions with an exercise specialist + 3 individual counselling sessions with an exercise specialist  CG: Usual care: received written materials about PA available through the American Cancer Society.  3 months | - Social Cognitive Theory;  - Trans- theoretical Model | - Daily caloric Intake (3-d diet record (i.e., 1 weekend and 2 weekdays) + Diet Analysis Plus software, version 7.0.1)  - PA: total daily activity counts, weekly minutes of moderate plus vigorous PA (GT1M accelerometer (Actigraph);  baseline, 3, 6 months | - Daily caloric intake showed a significant time effect (i.e., mean for baseline, 1,880.4; postintervention, 1,624.7; and 3 months postintervention, 1,624.6; F = 8.34; P < 0.001) with no significant effects of group (F = 0.03; P = 0.87) or group by time interaction (F = 0.35; P = 0.70).  - Daily PA counts: a significant group by time interaction (F = 4.28; P = 0.013) was noted.  -Similarly, weekly minutes of moderate plus vigorous activity and stage of motivational readiness showed significant group by time interactions (F = 3.51; P = 0.035; and F = 7.85; P < 0.001, respectively). |
| Kanera et al., 2016 | Multiple cancers at any stage, who have completed primary treatment at least 4 weeks and up to 56 weeks prior to initial participation (Breast 16,0%, colorectal 3,8%, lymphoma 17,0%, thyroid 13,7%, testes 12,3%, leukemia 6,6%, cervix 6,1%, brain 6,1%, others 18,4%) | Overall: 462;  IG: 231, 79,2% women, 55,6 ± 11,5 years;  CG: 231, 80,5% women, 56,2 ± 11,3 years; | IG: KNW, a web-based computer tailored intervention: Based on the screening questionnaire measuring several concepts such as Diet and PA + Dutch nutritional and PA guidelines, participants receive feedback on their dietary habits and their own level of PA and to which extent they reach the recommended level and then, they are encouraged to set a goal. Subsequently, dietary advice is given, personalized to the participant’s individual situation  CG: usual care/waiting list  6 months | - Integrated Model for Change (I-Change Model)  - Self-Regulation Theory | - Dietary behaviour: vegetable, fruit, whole grain bread, and fish consumption (8 items of the Dutch Standard Questionnaire on Food Consumption)  - PA (Short Questionnaire to Assess Health Enhancing Physical Activity (SQUASH));  baseline and at the 6-month follow-up | - No significant intervention effects were found for dietary variables after controlling for multiple testing (p>005).  - There were no significant differences in change over time concerning MPA between IG and CG after controlling for multiple testing (p>0.05). No significant results were found for Weekly days >30 min, Light PA min and Vigorous PA min (p>0.05). |
| Lee et al., 2018 | Colorectal, any stage, within one year of completion of main cancer treatment from the surgical/oncological departments | Overall: 223;  Group A: 55, 32,7% women, 63,2 ± 11,4 years; Group B: 56, 39,3% women, 65,9 ± 9,8 years; Group C: 56, 28,6% women, 66,6 ± 9,5 years; Group D: 56, 46,4% women, 64,9 ± 9,4 years; | IG: Group A (dietary and PA interventions), Group B (dietary only), Group C (PA only): individual face-to-face motivational interviews (two sessions for Groups A and B and one session for Group C), fortnightly motivational phone calls, mailed monthly stage-of-change matched educational pamphlets, mailed quarterly newsletters, and quarterly group meetings  CG: Group D (usual care): 5 pamphlets with general health advice that encouraged healthy lifestyles by eating a wide variety of food, more fruit and vegetables, increasing PA levels, quitting smoking and avoiding alcohol abuse.  12 months | - Theory of Planned Behaviour;  - Health Action Process Approach (HAPA) | Diet: Changes of dietary consumption, servings/day + Achieving behavioural targets (FFQ):  - Red and Processed Meat target: weekly intake of <5 servings, including <2 servings of processed meat,  - Refined Grains target: daily intake <2 servings,  PA: Changes of PA level, accumulated minutes of MVPA per week + Achieving behavioural targets (accelerometer):  - PA general health target: 30 minutes of MVPA 5 days a week  - PA cancer outcome target: 60minutes of MVPA 5 days a week;  Baseline, 6, 12, 18 and 24 months | Dietary interventions significantly:  - increased the odds of achieving the targets of consuming less processed meat at all time-points and refined grain at months 6 and 24  - reduced processed meat (all p<0.01) and refined grains (all p<0.01) consumptions.  - In the subgroup of 49 patients who had <300 minutes of MVPA per week at baseline, PA interventions did not significantly improve the two PA targets.  - However, patients who received the PA interventions had significantly larger increases in PA at months 6 (difference = 174.2, [34.7–313.7], p = 0.015) and 18 (179.0 [36.6–321.3], p = 0.014) than those who did not receive the PA interventions. |
| Campbell et al., 2009 | Colorectal | Overall: 266;  49,4%* women, 66,5±10,0* years;  TPC: 70 13,3%* women, 66,2±10,5* years;  TMI: 72 10,9%* women, 67,1±9,5* years; TPC+TMI: 58 12,5%* women, 66,9±9,8* years;  CG: 66, 12,7%* women, 66,6±10,1* years;  *sample consisted of cancer and non-cancer participants | IG:  - Tailored Print Communication (TPC): personalized computer-tailored newsletters focused on F&V consumption, PA, and follow-up surveillance as recommended by the participant's physician;  - Telephone Motivational Interviewing Intervention (TMI): four brief (20-min) MI calls;  -  TPC+TMI  CG: Generic Printed Health Information: two mailings of generic (nontailored) health information that was not related to the primary study outcomes.  12 months | - Social Cognitive Theory;  - Trans- theoretical Model;  - the principles of Motivational Interview | - Fruit&Vegetable Consumption: servings/day (36-item modified version of the Block food F&V: estimate one's intake with a 2-item screener + 35-item questionnaire (based on Block food frequency questionnaire (FFQ)) + average of both);  - Weekly PA: frequency (minutes/week) and MET hours/week of MVPA (modified version of 7-day PAR data)  Baseline and 12 months | - 35-item measure: No significant intervention effects were found for colorectal cancer survivors.  - 2 item measure: Statistically significant increases for all three intervention groups were found, compared to the control group, with both the TMI-only and combined groups showing an increase of more than one daily serving. The three intervention groups did not differ statistically from each other, however.  (CG: 4,3±2,0 vs TPC: 4,9±1,6 (p≤0,05) or TMI: 5,0±2,0 (p<0,01) or TPC+TMI: 5,2±2,4 (p<0,01))  - Averaging the 35-item and 2-item measures, no significant treatment differences among CRC survivors.  - None of the interventions produced significant effects on increasing PA among cancer survivors. |

CG: Control Group; IG: Intervention Group;

Appendix 4 – PA-only Trials

| **Authors** | **Cancer Type/Phase** | **Sample** | **Intervention** | **Theory** | **Outcomes** | **Results** |
| --- | --- | --- | --- | --- | --- | --- |
| Ungar et al., 2015 | Multiple cancers at any stage, receiving out-patient therapy (acute or maintenance therapy) or finished this therapy not longer than six months ago (Breast 32,8%, Colorectal 11,9%, Prostate 7,46%, others 47,8%) | **Overall:** 67;  **IG:** 35, 54,3% women, 56,69 ± 13,43 years;  **CG:** 32, 50% women, 54,09 ± 11,72 years | **IG - exercise intervention:** 1-h individual counselling session + booklet with behaviour change techniques; 3 weekly telephone calls (M = 14 min/call); 4-week practicing at home; and meeting with an exercise role model (physically active cancer patient) for walking/cycling together was encouraged  **CG - stress management intervention:** 1-h individual counselling session + booklet with stress-management techniques; 3 weekly telephone calls (M = 14 min/call); 4-week practicing at home; without PA information  4 weeks | HAPA-based counselling (enhancing self-regulation) + role model support. | PA: exercise min/week (self-reported Short QUestionnaire to ASsessHealth-enhancing PA (SQUASH) at T1, T2 and T3 and an accelerometer at T1 and T2.)  baseline (T1), 4 weeks (T2) and 14 weeks (T3). | - At 4 weeks after intervention, the intervention group had more PA than the control group: 45.7% of patients in the IG vs 18.8% in the CG increased their activity levels to meet PA guidelines (>150 min/week; χ 2 (1) = 5.51, p = 0.019).    - At 14 weeks after intervention, there were no significant differences (p = 0.225). |
| Hirschey et al., 2018 | Breast (stage Ia to IIb) being 2 months to 10-year status post-surgery, radiation, and chemotherapy | **Overall:** 58;  **IG:** 29, 100% women, 59 ± 10 years;  **CG:** 29, 100% women, 57 ± 12 years | **IG:** Exercise theory-guided booklet containing narrative messages, writing, and thinking activities intended to increase outcome expectations dimensions of importance, certainty and accessibility.  **CG:** Similar booklet focused on diet instead of exercise.  1 week intervention + 12 weeks follow up | Self-Efficacy Theory | PA:  - objective: Fitbit ®  - subjective: GLTEQ  baseline, 4-, 8-, and 12-weeks post intervention | There were positive significant differences in objectively measured steps between groups (IG: more 970 steps, p=0.0283), but not in subjective PA (p=0.268). |
| May et al., 2009 | Multiple cancers at any stage, medical treatment ≥3 months ago  (Breast 55,8; Haematological 16,6%; Gynaecological 11,6&; Urogenital 5,5%; lung 2,7; other 6,2% | **Overall**: 147, 83,7% women, 48,8 ± 10,9 years; **IG: 76**, 86,8% women, 47,8 ± 10,5 years; **CG:** 71, 80,3% women, 49,9 ± 11,3 years | **IG:** Physical training + cognitive-behavioural training  **CG:** Physical Training: supervised exercise programme: aerobic and resistance exercise, and group sports  12 weeks | Self-Management | PA: Physical Activity Scale for the Elderly (PASE)  baseline, 12 weeks | Changes in PA from baseline to post-intervention were not significantly different between groups (p>0.05) |
| Rogers et al., 2014 | Breast (DCIS, stage I-IIIA) not currently receiving or planning to receive chemotherapy or radiation therapy. | **Overall**: 222, 100% women, 54,4 ± 8,5 years;  **IG: 110**, 100% women, 54,9 ± 9,3 years;  **CG:** 112, 100% women, 53,9 ± 7,7 years | **IG:** Six discussion group sessions + 12 supervised exercise sessions + multiple home-based exercise sessions beginning in the third week + three face-to-face update counselling sessions  **CG:** Usual care: printed American Cancer Society materials describing PA recommendations for cancer survivors  3 months | Social Cognitive Theory | weekly minutes of ≥moderate intensity PA (MTI/ActiGraph accelerometer + GLTEQ)  baseline, immediately post-intervention (month 3; M3), and 3 months post-intervention (month 6; M6) | Between group mean differences in PA was statistically significant at M3 (accelerometer, +41 weekly minutes, p = 0.010; self-report, +93 weekly minutes, p=0.001) and remained statistically significant at M6 for self-reported PA (+74 weekly minutes, p=0.001), but not for accelerometery. |
| McGinnis et al., 2021 | Multiple cancers at any stage, currently receiving or within six-months of receiving active cancer treatment (Breast 39,3; prostate, 7,1%; ovarian, 7,1%; haematological, 17,9%; other, 28,6%) | **Overall:** 33, 63,6% women, 54,3 ± 12,4 years; | **IG:**  Exercise Program + PA Behaviour Change Counselling  **CG:**  Exercise Program  3 months | Social Cognitive Theory | PA (Adapted version of the GLTEQ)  Baseline and post-program (3M) | Intervention Group reported an 81.3% increase in minutes per week of MVPA (M = 108.33 ± 166.5 min), compared to a 16.6% increase (M = 38.57 ± 114.6) in the control group. Intervention group 67% (n = 4) reported an increase of ≥ 60 min per week of MVPA, compared to 25% (n = 2) participants in the control group.  p-value NA |
| Courneya et al., 2016 | Colon (stage II and III) who received adjuvant chemotherapy within the past 2–6 months, | **Overall:** 211;  **IG:** 106, 57% women, <65 yrs (67%); ≥65 yrs (33%);  **CG:** 10, 56% women, <65 yrs (68%); ≥65 yrs (32%); | **IG:** Behaviour support sessions + Supervised exercise sessions + exercise guidebook developed specifically for colon cancer  **CG:** general health education materials promoting PA and healthy nutrition and standard surveillance follow-up.  3 years | Theory of Planned Behaviour | Sel reported recreational PA  (Total Physical Activity Questionnaire - TPAQ (converted to MET-hours/week))  Baseline, 6, 12, 18, 24, 30 and 36 months | The intervention group reported a significant increase in PA of 15.6 MET-hours/week from baseline to 1 year compared to the control group with an increase of 5.1 MET-hours/week (p=0.002). |
| Bélanger et al., 2014 | Multiple cancers at any stage/phase of treatment  (Breast (16.0%), colorectal, lymphoma (17.0%), thyroid (13.7%), testes (12.3%), leukaemia (6.6%), cervix (6.1%), brain (6,1%), colorectal (3,8%) others (18,4%) | **Overall:** 212, 60,8% women, 18-29 yrs (25,9%); 30-39 yrs (74,1%);  **IG**: 106, 60,4% women, 18-29 yrs (24,5%); 30-39 yrs (75,5%);  **CG**: 106, 61,3% women, 18-29 yrs (27,4%); 30-39 yrs (72,6%); | **IG:** Thrive to Survive Guidebook: with information about the protective effect of PA against chronic disease, tips on how to make PA enjoyable, how much PA is recommended, how to determine PA intensity, and practical tips such as how to dress for the weather. The Guidebook included participant-centred activities designed to facilitate participant engagement in the information as well as control over PA behaviour, including instructing the reader to scan their current physical environment for opportunities to be physically active, a time management worksheet, information about goal setting, and a PA tracking sheet. Throughout the Guidebook there were motivational quotes from YACS with an accompanying picture of the person performing an activity, as well as motivational quotes from oncologists and exercise specialists.  **CG:** Received Canadian PA Guidelines (CPAG).  Does not specify intervention’s duration | Theory of Planned Behaviour | PA (Modified LSI from GLTEQ)  Baseline, 1, and 3 months | No differences at 1 and 3 months for all sample (p>0.05). But participants who reported ≤300 PA minutes/week and participate in the intervention group had significant differences in total PA at 3 months: mean change of +135 minutes/week vs +69 minutes/week on the CG (p=0.028); but not at 1 month |
| Vallance et al., 2015 | Breast (stages I-IIIA), scheduled to receive neoadjuvant or adjuvant chemotherapy | **Overall**: 95, 100% women, 52,8 ± 9,8 years;  **IG:** 49, 100% women, 52,8 ± 9,6 years;  **CG:** 46, 100% women, 52,9 ± 10,1 years | **IG:** PROACTIVE PA resource kit: PA print materials, a step pedometer, and a step logbook  **CG:** Generic two-page public health PA resource: Canada's Physical Activity Guide to Healthy Active Living for Healthy Adults or Canada's Physical Activity Guide for Older Adults.  4-6 months (during chemo) | Theory of Planned Behaviour | - Objective walking behaviour: Pedometer steps (3-day step test using the StepsCount SC-01 pedometer);  - Self-reported PA (LSI from the GLTEQ);  Baseline (prior to second chemotherapy administration) and at post intervention (between 3 and 4 weeks after the last chemotherapy administration). | Intervention was not statistically superior to a standard recommendation for daily average pedometer steps (P = 0.22), light intensity PA minutes (P = 0.70), moderate intensity PA minutes (P= 0.90), vigorous intensity PA minutes (P = 0.93) and total MVPA minutes (P = 0.90). |
| Kong et al., 2021 | Breast (stage I to III) who were planning to undergo radiation therapy (RT) after surgery | **Overall:** 152;  **IG**: 76, 100% women, 47,3 ± 8,5 years;  **CG**: 76, 100% women, 46,8 ± 7,6 years; | **IG:** Wearable Activity Tracker (WAT) + counselling* (weekly face-to-face by a exercise physiologist) + educational booklets  **CG:**  counselling* (weekly telephone by an exercise physiologist) + educational booklets  during the 5-week radiotherapy treatment (RT) period | Trans- theoretical Model | - Self-reported Leisure Time Physical Activities levels (Global Physical Activity Questionnaire (GPAQ));  before RT, immediately after RT, and 3 and 6 months after completion of RT. | - The IG had increased relative change in self-reported LTPA (102.8) compared with the CG (57.8) immediately after RT compared to baseline. Although the relative changes of self-reported LTPA of the IG were higher at three and six months after the end of RT compared to in the CG, the results were not significant.  - The mean average daily step count of the IG was 9351.7, which increased to 11,592.2 during RT and 12,240.1 after RT |
| Weiner et al., 2019 | Breast (diagnosed less than 5 years prior to study enrolment, had completed chemotherapy and/or radiation treatment) | **Overall:** 87;  **IG**: 43, 100% women, 58,2 ± 11,4 years;  **CG**: 44, 100% women, 56,2 ± 9,2 years | **IG:** face-to-face meeting with a trained interventionist (who used motivational interviewing techniques to help each participant set a specific, personalized PA goal and an action plan to gradually increase their activity) + received a Fitbit ® + two 20-min phone calls at the 2- and 6-week time points (to review Fitbit data and discussing progress toward the goal) + twice-weekly emails with theory-based content and reminders to wear and sync their Fitbit  **CG:** Waitlist wellness contact control condition received standardized emails every 3 days on women’s health topics (e.g., healthy eating, stress management, and general brain health).  12 week | - Social Cognitive Theory;  - Control Theory | PA: MVPA, LPA (ActiGraph GT3X+ accelerometer)  baseline and 12 weeks | No differences between groups in LPA (p = 0.48) but positive significant differences in accelerometer-measured MVPA: mean increase 14.2 min per day (SD = 13.9) in the IG vs. − 0.7 min per day (SD = 9.7) in the CG (p < 0.001). |
| Webb et al., 2019 | Multiple cancers at any phase (breast 38.2%, prostate 6.8%, colorectal cancer 13.0%, others 42.0%) | **Overall**: 207, 73,9% women,  0-44 yrs (14,5%);  45-64 yrs (61,8%);  ≥65 yrs (23,7%);  **IG:** 104, 72,1% women,  0-44 yrs (14,4%);  45-64 yrs (65,4%);  ≥65 yrs (20,2%);  **CG:** 103, 75,7% women,  0-44 yrs (14,6%);  45-64 yrs (58,3%);  ≥65 yrs (27,2%); | **IG:** printed components and Internet tools and e-newsletters influenced by the stage of PA behaviour change model with content tailored by prediagnosis levels of PA, age and gender  **CG:** standard letter recommendation  24 weeks (▪Intervention: 12 weeks + 12 weeks follow up ▪Control: 12 weeks standard letter + 12 weeks intervention) | - Social Cognitive Theory;  - Theory of Planned  Behaviour; | PA: GLTEQ  12 and 24 weeks | The intervention arm reports a mean PA improvement score of 9.58 (23.14) over 12 weeks, compared with 2.61 (24.10) in the control arm (p = 0.04).  At 12 weeks: IG: 35.57 ±23.71 vs CG: 31.31± 22.65; p<0,05. |
| Short et al., 2015 | Breast (stage 0-4), who completed active cancer treatment | **Overall**: 330, 100% women, 55 years; **Tailored IG:** 109, 100% women, 56 years; **Targeted IG**: 110, 100% women, 55 years;  **CG:** 111, 100% women, 55 years | **IG:**  **Tailored-print intervention group** received three Social Cognitive Theory-based computer-tailored newsletters over a 12-week period (6 weeks apart);  **Targeted-print intervention group** received a copy of the Theory of Planned Behaviour-based booklet ‘Exercise for health: An exercise guide for breast cancer survivors’;  **CG:** Received the brochure ‘An active way to better health’ describing the national PA guidelines for Australian adults  12 week | - Social Cognitive Theory;  - Theory of Planned Behaviour | - Self-reported PA and  - Meeting the PA guidelines for aerobic and resistance-based activity (adapted version of the LSI from the GLTEQ);  - Mean daily steps (Pedometer);  baseline, 4 months | Allocation to the tailored intervention significantly reduced the odds of not doing any resistance-based PA (p < 0.01) relative to the control group and  - increased the odds of meeting the resistance training guidelines.  - Meeting aerobic guidelines was not significantly different between groups (p>0.05).  - There were no other significant intervention effects.  -Aerobic: n aumentou nem guidelines  - Resistance: s s |
| Pinto et al., 2013 | Breast, stage 0-IV (completed primary and adjuvant treatment for breast cancer ≤ 5 years since treatment completion) | **Overall**: 192, 100% women, 60,0 ± 9,9 years; **IG:** 86, 92% women, 59,5 ± 9,7 years; **CG:** 106, 93% women, 62,8 ± 11,1 years | **IG:** health care provider advice for PA + 12 weeks of telephone counselling + Monthly PA calls for 3 months  **CG:** health care provider advice for PA + 12 weeks of contact control + Monthly calls for 3 months  6 months | - Social Cognitive Theory  - Trans-theoretical Model; | PA (7-day PAR)  baseline, at 3 months, 6 and 12 months. | Intervention participants had more MPA than the control group at both 3 months (59,70 vs 30,82; p =0.048) and 6 months (56,64 vs 32,16; p = 0.032), but this beneficial telephone counselling effect dissipated at 12 month (p = 0.574). |
| Pinto et al., 2015 | Breast (stage 0-3) who had completed surgery | **Overall:** 76, 100% women, 55,62 ± 9,55 years;  **IG**: 39, 100% women, 55,64 ± 8,59 years;  **CG**: 37, 100% women, 55,59 ± 10,59 years; | **IG:** PA + Reach to Recovery program: telephone-based PA counselling, a pedometer (Digiwalker) and a heart rate monitor + Reach to Recovery program informational booklets + 12 exercise tip sheets that focused on PA topics  **CG:** Reach to Recovery program: 12 calls during which was administered the Weekly Symptom Questionnaire + Reach to Recovery program informational booklets.  12 weeks | - Social Cognitive;  - Trans-theoretical Model | Self-reported MVPA (7-day PAR);  Mean minutes of MVPA (Accelerometer (Actigraph GT3X))  baseline, 12, 24 weeks | For self-reported PA, there were significant group differences favouring the intervention group, compared to the control group in minutes of MVPA at 12 weeks (129,5±73,4 vs 25,0 ± 67,4; p<0.001) and at 24 weeks (98,4±83,2 vs 63,9 ± 82,9; p=0.03).  For accelerometery significant group differences favouring the intervention group, compared to the control group in minutes of MVPA at 12 weeks (70,3±65.9 vs 16.5±31.9; p<0.01) and at 24 weeks (54,6±81,6 vs 13,4 ± 35,2; p<0.01) |
| Golsteijn et al., 2018 | Colorectal, Prostate undergoing curative treatment or completed primary treatment up to one year ago. | 478;  **IG**: 249, 14,9% women, 66,55 ± 7,07 years;  **CG**: 229, 10,9% women, 66,38 ± 8,21 years; | **IG:** pedometer + computer-tailored PA advice, both Web-based via an interactive website and with printed materials.  **CG:** usual care waiting-list  4 months | - Social Cognitive Theory;  -Trans-theoretical Model;  - HAPA;  - I-Change Model;  -Health Belief model; - goal setting theories;  - theories of self-regulation and the Precaution Adoption Process Model: targets pre-motivational constructs | Objective MVPA (ActiGraph GT3X-BT accelerometer) - baseline, 6 months;  Self-reported MVPA (SQUASH) - baseline, 3, 6 months; | At 3 months, participants in the OncoActive group improved their PA significantly in terms of both MVPA (B = 133.55, p = 0.04) and days with at least 30 min of PA (B = 0.86, p < 0.001). At 6 months, results indicate significant improvements in self-reported PA (MVPA: B = 267.17, p < 0.001; Days ≥30 min PA: B = 0.98, p < 0.001). ActiGraph assessed MVPA also increased significantly (MVPA: B = 44.60, p = 0.006), whereas the increase in ActiGraph assessed days ≥30 min PA was borderline significant (B = 0.38, p = 0.05). |

CG: Control Group; IG: Intervention Group;

| **Study** | **Design** | **Blinding** | **Selection bias** | **Drop-outs** | **Confounders** | **Data collection** | **Data analysis** | **Report** | **Global rating** |
| --- | --- | --- | --- | --- | --- | --- | --- | --- | --- |
| **Diet-only** |  |  |  |  |  |  |  |  |  |
| M. Miller et al., 2020 | Strong | Weak | Weak | Moderate | Weak | Strong | Moderate | Strong | Weak |
| Parsons et al., 2020 | Strong | Weak | Weak | Strong | Weak | Strong | Strong | Strong | Weak |
| **PA-only** |  |  |  |  |  |  |  |  |  |
| Webb et al., 2019 | Strong | Moderate | Weak | Strong | Strong | Strong | Strong | Strong | Moderate |
| Hirschey et al., 2018 | Strong | Weak | Weak | Strong | Strong | Strong | Moderate | Strong | Weak |
| Pinto et al., 2013 | Strong | Moderate | Weak | Strong | Strong | Strong | Strong | Strong | Moderate |
| Ungar et al., 2016 | Strong | Weak | Weak | Strong | Weak | Strong | Moderate | Strong | Weak |
| Weiner et al., 2019 | Strong | Weak | Weak | Moderate | Strong | Strong | Strong | Strong | Weak |
| Kong et al., 2021 | Strong | Weak | Weak | Strong | Strong | Strong | Strong | Strong | Weak |
| Courneya, 2010 | Strong | Weak | Weak | Strong | Weak | Strong | Moderate | Strong | Weak |
| Bélanger et al., 2014 | Strong | Weak | Weak | Strong | Strong | Strong | Strong | Strong | Weak |
| Rogers et al., 2014 | Strong | Moderate | Weak | Strong | Strong | Strong | Strong | Strong | Moderate |
| Vallance et al., 2016 | Strong | Weak | Weak | Strong | Strong | Strong | Strong | Strong | Weak |
| McGinnis et al., 2021 | Strong | Moderate | Weak | Strong | Weak | Strong | Moderate | Moderate | Weak |
| (May et al., 2009 | Strong | Moderate | Weak | Strong | Strong | Strong | Strong | Moderate | Moderate |
| Short et al., 2015 | Strong | Moderate | Weak | Strong | Moderate | Strong | Strong | Strong | Moderate |
| Pinto et al., 2015 | Strong | Weak | Weak | Strong | Moderate | Strong | Moderate | Moderate | Weak |
| Golsteijn et al., 2018 | Strong | Weak | Weak | Moderate | Strong | Strong | Strong | Strong | Weak |

Appendix 5 - Risk of bias assessment

| **Study** | **Design** | **Blinding** | **Selection bias** | **Drop-outs** | **Confounders** | **Data collection** | **Data analysis** | **Report** | **Global rating** |
| --- | --- | --- | --- | --- | --- | --- | --- | --- | --- |
| **Multiple-behavior** |  |  |  |  |  |  |  |  |  |
| M. Campbell et al., 2009 | Strong | Moderate | Weak | Strong | Strong | Strong | Moderate | Moderate | Moderate |
| Sturgeon et al., 2016 | Strong | Moderate | Weak | Strong | Weak | Strong | Moderate | Strong | Weak |
| Lee et al., 2018 | Strong | Moderate | Weak | Strong | Strong | Strong | Strong | Strong | Moderate |
| Hawkes et al., 2013 | Strong | Moderate | Weak | Strong | Strong | Strong | Strong | Strong | Moderate |
| Gruenigen et al., 2008 | Strong | Weak | Weak | Strong | Moderate | Strong | Strong | Strong | Weak |
| Kanera et al., 2016 | Strong | Weak | Weak | Strong | Strong | Strong | Strong | Strong | Weak |
| Mosher et al., 2012 | Strong | Weak | Weak | Strong | Strong | Strong | Strong | Strong | Weak |
| Rogers et al., 2009 | Strong | Weak | Weak | Strong | Weak | Strong | Strong | Strong | Weak |
| Gruenigen et al., 2012 | Strong | Weak | Weak | Moderate | Strong | Moderate | Strong | Strong | Weak |

Appndix 5 continued

Appendix 6 - SURE Checklist

A - Identification, selection and appraisal of studies

A1) Were selection criteria reported?

Yes

A2) Was the search comprehensive?

Partially

A3) Is the review up-to-date?

Yes

A4) Was biased selection of articles avoided?

Yes

A5) Were appropriate criteria used to assess the risk of bias?

Yes

A6) Overall identification, selection and appraisal of studies

Reliable

B - Analysis of the findings

B1) Were characteristics and results of included studies reliably reported?

Yes

B2) Were methods used to analyse the findings reported?

Yes

B3) Was the extent of heterogeneity described?

Yes

B4) Were the findings combined (or not combined) appropriately?

Yes

B5) Were factors that could explain heterogeneity explored?

Not applicable

B6) Overall analysis of findings

Reliable

C - Overall assessment of the reliability of the review

C1) Other considerations

No other quality issues identified

C2) Overall reliability of the review

Reliable: This is a good quality systematic review with only minor limitatio
